# The illusion of conformity: when independent learning mimics social influence

**DOI:** 10.1093/beheco/arag122

**Published:** 2026-10-16

**Authors:** Frédérique Dubois

**Affiliations:** Département des Sciences Biologiques, Université de Montréal, 1375 Avenue Thérèse-Lavoie-Roux, Montréal, Québec H2V 0B3, Canada

**Keywords:** behavioral differences, competition-predation tradeoff, conformity, cost of deviation, individual trial-and-error learning, oddity effect

## Abstract

Although individuals tested in isolation often show substantial behavioral variation, they tend to behave more uniformly in groups, a pattern commonly attributed to social conformity (ie, a form of social learning in which individuals preferentially adopt the most common trait). However, evidence for social conformity in nonhuman animals remains debated, as group-level convergence can also arise from independent learning, where individuals adopt the trait maximizing their fitness. Such learning can generate either adaptive phenotypic convergence or pseudo-conformity when aligning with the group's average strategy provides a selective advantage (eg, by reducing predation risk). The likelihood of these outcomes is shaped by ecological and social factors, yet their relative contributions remain poorly understood, as most studies rely on simplified laboratory conditions. This study explores the conditions under which conformist-like patterns can emerge solely from individual learning, and how costs of deviating from the group strategy and tradeoffs between different selective pressures influence their evolution. I developed an evolutionary model in which individuals sequentially adjust their exploratory behavior through a trial-and-error learning process. At each learning step, an individual explores 2 small behavioral deviations around its current exploration level and retains the variant that yields the highest payoff under the prevailing ecological conditions. The results show that the likelihood of pseudo-conformity depends on the interplay among ecological conditions, group composition and behavioral flexibility, and is particularly favored when predation risk and the energetic costs of escaping predator attacks are high. Moreover, under some conditions, pseudo-conformity may maintain behavioral diversity at the population level by allowing different subgroups to converge on and stabilize around distinct behavioral phenotypes. Overall, these findings underscore that measuring traits in isolation may misrepresent both behavioral variation and evolutionary dynamics.

## Teaser

Animals often behave differently when alone but become more similar in groups. This is usually explained by social conformity, where individuals copy the most common behavior. However, similar group patterns can also arise without copying, simply because each individual learns through trial and error which behavior works best. In this study, I used a model to explore when such “conformist-like” patterns can emerge from individual learning alone and discuss the resulting evolutionary consequences.

## Introduction

Individuals within the same population typically display considerable variation in phenotypic and physiological traits. However, despite this inherent diversity, they tend to behave similarly or make uniform decisions in the presence of conspecifics, a phenomenon that may be attributed to social conformity. Social conformity refers to the social learning bias whereby individuals preferentially adopt the traits most frequently expressed by other group members, thereby aligning with the population norm (Laland 2004; Kendal and Watson 2023). The classic line judgment experiments by Asch (1956) were pioneering in demonstrating that social pressure from a group can lead humans to conform to a majority opinion, even when that opinion contradicts their own perception of reality. In Asch's experiments, subjects were asked to identify which of the 3 lines had the length of a reference line, a simple task with an obvious correct answer. Therefore, when making this decision in isolation (ie, without social influence), participants gave the incorrect answer <1% of the time. Conversely, when surrounded by actors who unanimously gave the same incorrect answer, over one-third of participants conformed and chose the wrong line (Asch 1956). Since these pioneering studies, a substantial body of research has provided additional empirical evidence that humans tend to adopt the most common traits, whether discrete or continuous (eg, Baron et al. 1996; Bond and Smith 1996; Campbell-Meiklejohn et al. 2010; Jayles et al. 2017). By contrast, empirical evidence in favor of social conformity in nonhuman animals remains controversial, since alternative learning processes often provide equally plausible explanations (van Leeuwen et al. 2015).

Experimental research on nonhuman animals has shown that individuals often modulate their behavior in response to social context, leading to reduced interindividual behavioral variation when individuals are tested in groups compared with in isolation (Magnhagen and Staffan 2005; Magnhagen and Bunnefeld 2009; Schuett and Dall 2009; King et al. 2015; McDonald et al. 2016; Burns et al. 2017; Guayasamin et al. 2017; Fürtbauer and Fry 2018; Munson et al. 2021; Skinner et al. 2024). These socially induced shifts are commonly interpreted as evidence of social conformity, whereby individuals modify their behavior in response to the social context, to align with the trait most commonly expressed by others. However, similar group-level patterns may also result from independent trial-and-error learning, whereby all individuals converge towards the same phenotype without relying on social learning. This process may result from adaptive phenotypic convergence or pseudo-conformity. Specifically, shared environmental cues or constraints (eg, resource distribution, predation risk, or environmental heterogeneity) can lead individuals to independently adopt the behavior best suited to local ecological conditions. Alternatively, aligning with the group's average strategy may provide a selective advantage, for instance by reducing predation risk, thereby generating pseudo-conformity. A key objective of the present study is to evaluate whether, and under what conditions, conformist-like patterns can emerge purely from individual trial-and-error learning, whereby each individual independently adopts the trait value or option that maximizes its own fitness. Moreover, although the likelihood of these outcomes is shaped by both ecological and social factors, their relative contributions remain poorly understood. This is because most conformity experiments have been conducted in simplified laboratory settings, typically involving interactions with only one conspecific, thereby limiting our understanding of how environmental and social factors jointly influence the expression of interindividual variation in more complex social contexts.

The fact that individuals align their behavior with that of other group members is generally explained by the existence of deviation costs (ie, costs associated with adopting a trait that differs from those adopted by others) that promote conformity via positive frequency-dependent selection. Deviation costs may be social, informational or ecological. For example, substantial evidence in humans shows that deviant individuals incur negative social consequences, such as exclusion or sanctions, that drive normative conformity to group decisions, even when those decisions are arbitrary or incorrect (Asch 1956; Baron et al. 1996; Kundu and Cummins 2013), which can, in turn, reduce individual fitness. Conformity can also emerge though informational processes when trait frequency signals adaptiveness (ie, when the most common trait is also the most advantageous). In such cases, deviant individuals face an elevated risk of adopting trait variants that are less well suited to local conditions (Boyd and Richerson 1985; Laland 2004). Finally, ecological costs of deviation may favor conformity when, for example, individuals whose behavior deviates from that of the group (ie, “odd” individuals; Milinski 1977; Theodorakis 1989) are more frequently targeted by predators than those exhibiting more common phenotypes (eg, Landeau and Terborgh 1986; Penry-Williams et al. 2018; Aivaz et al. 2020).

Thus, the costs of deviation should a priori consistently favor the emergence of conformist-like patterns. However, because a single trait usually affects multiple fitness components that may be subject to opposing selective pressures, such tradeoffs could, in some cases, prevent behavioral convergence and instead contribute to the maintenance of interindividual differences. For instance, several behavioral traits (eg, boldness, activity, exploration, sociability) are subject to a tradeoff between predation and competition (eg, Sih 1987; Anholt and Werner 1995; Réveillon and Becks 2024): bolder, more exploratory and/or more active individuals are better at monopolizing resources (Herborn et al. 2010; Pereira et al. 2024) but usually suffer an increased risk of being detected by predators (Jones and Godin 2010). Under conditions of intense competition, the predation-related benefits of behavioral alignment may therefore be surpassed by the advantages of expressing trait values exceeding those of other group members, making pseudo-conformity less likely. An important aim of the study, therefore, is to better understand how environmental and social factors jointly shape reliance on pseudo-conformity, a process with potentially broad implications for both humans and nonhuman animals (Henrich and Boyd 1998; Molleman et al. 2013). To investigate these mechanisms, I developed an evolutionary model in which each group member sequentially adjusts its level of exploration through a trial-and-error process: at each time step, individuals in turn slightly modify their current level of exploration and retain the new behavior only if it improves their payoff. This payoff represents their net energetic gain and is calculated as the balance between the energy acquired through resource exploitation and the energetic costs associated with exploration and escaping predator attacks. For each evolutionary scenario considered, I simulated 100 groups with varying initial compositions (in terms of each member's level of exploration expressed when isolated from others) and explored the conditions under which all group members converged towards the same trait value. When convergence occurred, I determined whether it arose from pseudo-conformity (where individuals iteratively adopt a trait value corresponding to the mean of the trait expressed by the other group members in isolation) or adaptive phenotypic convergence (whereby all individuals adopt the phenotype best suited to the prevailing ecological conditions). Although both pseudo-conformity and adaptive phenotypic convergence lead group members to all adopt the same behavior which, in principle, tends to reduce diversity, they differ markedly in their broader implications. Unlike adaptive phenotypic convergence, pseudo-conformity may actually contribute to the maintenance of between-group differences (Boyd and Richerson 1988; Henrich and Boyd 1998), thereby mitigating some of the negative consequences of convergence for population resilience. Finally, because tradeoffs among fitness components may constrain individuals’ ability to align their behavior with that of others, particularly when individuals face limits on behavioral flexibility, I tested whether the model's conclusions remained robust under constraints on behavioral flexibility. Though I modeled the individuals’ energetic gain according to their exploration level, the conclusions of the model can be extended to other behavioral traits (eg, aggression, sociability, or boldness) that affect multiple fitness components (eg, Haave-Audet et al. 2022).

## The model

The model (with all parameters detailed in Table 1) considers a group of *N* individuals competing for limited resources while facing predation risk. Each individual *i* is defined by an exploration level $e_{i}$, randomly drawn from a uniform distribution between 0 and 1 at the start of the simulation. Each simulation lasts 80 steps, which is sufficient to capture the resulting behavioral patterns, as extending the simulations beyond 80 steps does not qualitatively alter the outcome categories; at each step, individuals update their exploratory behavior sequentially following a trial-and-error learning process, in order to maximize their energetic gain (used as a proxy for fitness). The energetic gain of an individual corresponds to the difference between the energy acquired through resource exploitation and the energetic costs associated with exploration and escaping predator attacks, with benefits and costs being directly influenced by the exploratory behavior of all group members.

**Table 1:** List and meaning of the symbols used in the model.

Symbol	Meaning	Tested values
*N*	Group size	5, 10, 15, 20
*R*	Total amount of available resources	5, 20, 35
$c_{m}$	Energetic cost of exploration	1, 3, 5
$c_{e}$	Energetic cost of escaping a predator attack	3, 6, 9
$\delta_{\max}$	Maximum probability that the group is detected by a predator	0.2, 0.5, 0.8
odd	Oddity effect (the lowest values indicate a higher risk for odd individuals to be the target of predators)	0.1, 0.6, 1.1
*a*	Strength of the tradeoff between exploration and vigilance	0.2
flex	Range of exploration values that an individual can express around its baseline value measured in isolation	0.3, 1
*I*	Quantity of resources obtained by individual *i* (see Equation 2)	…
*K*	Expected number of individuals that are not vigilant at any given time (see Equation 4)	…
*δ*	Probability that the group is detected by a predator (see Equation 3)	…
*α*	Probability that individual *i* is the target of a predator's attack (see Equation 5)	…

For symbols that correspond to model parameters, the tested values are indicated.

Explicitly, the energetic gain of individual *i*, $W(e_{i})$, consists of 3 components (each of which is described in detail below) and is defined as:


(1) $$W(e_{i})=I-c_{m}\times e_{i}-c_{e}\times \delta \times \alpha$$

where *I* denotes the energy acquired through resource exploitation, $c_{m}$ the metabolic cost of exploration (eg, Careau and Garland 2012), $c_{e}$ the amount of energy expended to escape a predator's attack, *δ* the probability that the group is detected by a predator and *α* the probability of being the target during a predator attack (Fig. 1a).

**Figure 1:**
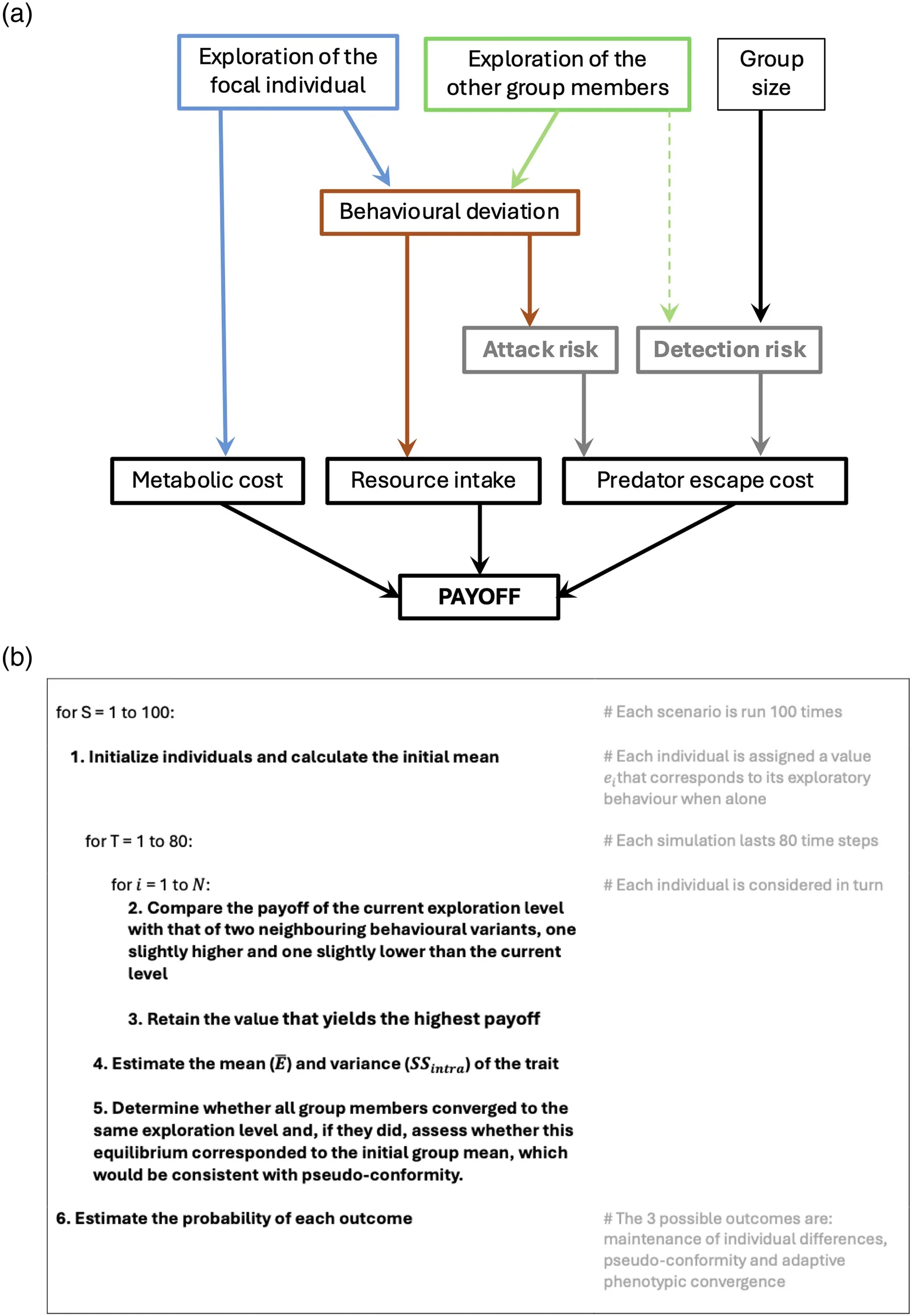
a) Schematic illustration of the competition–predation tradeoff shaping the 3 components of an individual's energetic gain. Individuals whose behavior deviates markedly from the group mean are more likely to be targeted by predators, thereby incurring an escape cost, but may secure a greater share of resources when their level of exploration exceeds that of their group mates. However, higher exploration levels also entail greater metabolic costs. b) Pseudocode summarizing the main steps of the learning algorithm.

### Component related to competition

I assume that the total amount of available resources is *R*, and that the quantity of resources obtained by an individual depends on both its exploratory behavior and the number and behavior of the other group members. There is empirical evidence, indeed, that bolder and more exploratory individuals are more likely to encounter profitable foraging opportunities and high-quality prey types (eg, Herborn et al. 2010; Pereira et al. 2024). Therefore, when all group members display the same exploration level, resources are shared equally among them. In contrast, when individuals differ in their exploration level, those exhibiting greater exploration secure a larger share of the resource, in proportion to the extent of their behavioral deviation from other competitors. If *K* represents the sum of the exploration levels of all group members, the quantity of resources obtained by individual *i* (whose level of exploration is $e_{i}$) can thus be estimated as:


(2) $$I=\frac{e_{i}}{K}\times R$$

### Components related to predation

I assume that predation risk of individual *i* is influenced by group size and the extent of the behavioral difference between its exploration behavior and the average exploration behavior of other group members $\bar{E}$, with $\bar{E}=\sum_{\;j=1}^{N-1}e_{j}/(N-1)$. Larger groups are more likely to be detected by a predator, though this effect may be mitigated by an increase in collective vigilance. Concretely, the risk that the group is detected and attacked by a predator is estimated as:


(3) $$\delta =\delta_{\max}\times (\;1-e^{-aK})$$

Because an individual's level of exploration varies between 0 and 1, it represents the proportion of time devoted to exploration, which is incompatible with vigilance. The parameter *K* can therefore be interpreted as the expected number of individuals that are not vigilant at a given time and is calculated, as above, as: $K=\sum_{i=1}^{N}e_{i}$. Thus, in (Equation 3), $\delta_{\max}$ denotes the maximum predation risk, that is the asymptotic value approached by *δ* as K increases. The parameter *a* determines how rapidly predation risk increases with *K* and hence reflects the strength of the tradeoff between exploration and vigilance (eg, Boujja-Miljour et al. 2018; Mettke-Hofmann 2022). Accordingly, low values of *a* result in a more gradual increase in predation risk with increasing *K*, whereas high values of *a* produce a steeper increase.

Once the group is detected by a predator, the predator may attack several prey with the group. Therefore, the probability of an individual being targeted (ie, *α*) is estimated independently for each group member and depends on the absolute difference between an individual's exploratory behavior and the mean behavior of the other group members, as well as on the strength of the oddity effect (odd), with:


(4) $$\alpha =d+(1-d)\times \frac{\left|e_{i}-\bar{E}\right|}{\mathrm{odd}+|e_{i}-\bar{E}|}$$

In Equation (4), $d=\frac{1}{N}$ corresponds to an individual's risk of being attacked, which is diluted among all group members when they adopt the same exact behavior (ie, when $|e_{i}-\bar{E}|=0$), while the parameter odd (with odd > 0) reflects the oddity effect: the lower the value of this parameter, the odder individuals become targets of predator attacks.

### Analysis

The main steps of the algorithm are indicated in the pseudocode (Fig. 1b). At the beginning of every simulation, the model randomly assigns at each group member a level of exploration that reflects its behavior when tested in isolation. Then, at each of 80 consecutive learning steps, the model allows all group members to sequentially adjust their behavior to maximize their energetic gain through a trial-and-error learning process. Specifically, at each learning step, individual *i* evaluates the energetic payoff (Equation 1) associated with its current exploration level as well as with 2 neighboring behavioral variants, one slightly lower (−0.01) and one slightly higher (+0.01) than the current level. The individual then adopts the behavioral variant that yields the highest payoff. Behavioral adjustments are restricted to the interval $\left[e_{i}-\mathrm{flex},e_{i}+\mathrm{flex}\right]$ (bounded between 0 and 1), where flex represents the individual's behavioral flexibility.

Once all individuals have adjusted their behavior a last time, I estimate the mean exploration level adopted by the *N* group members ($\bar{E}$) and the within-group variance among individuals (SS_intra) with:


(5) $$SS_{\mathrm{intra}}=\frac{\sum_{i=1}^{N}\;{\left(e_{i}^{*}-\bar{E}\right)}^{2}}{N}$$

Based on the within-group variance, I consider that all individuals have converged towards the same trait value if $SS_{\mathrm{intra}}<0.002$, or not otherwise. If convergence occurred, I then determined whether it resulted from pseudo-conformity by assessing whether the group converged to its initial mean trait value. Convergence was classified as pseudo-conformity when the absolute difference between the group's initial mean exploration level and the final trait value shared by all group members was smaller than 0.05. For a given ecological scenario (ie, set of parameter values), I generated 100 replicates and used the results of those replicates to estimate the frequency of each outcome (ie, persistence of individual differences in exploration, convergence toward a common trait through pseudo-conformity, or convergence through adaptive phenotypic adjustment).

I varied group size (*N*), the amount of available resources (*R*), the metabolic cost of exploration ($c_{m}$), the risk of predation ($\delta_{\max}$), the cost of escaping a predator attack ($c_{e}$), as well as the oddity effect (see Table 1), for a total of 972 distinct ecological scenarios.

To investigate whether group composition also influenced the likelihood of pseudo-conformity, I then selected ecological scenarios that yielded an intermediate frequency of pseudo-conformity, and for each scenario, I used binary logistic regressions based on 100 replicate simulations to test whether the mean exploration level of individuals in isolation predicted the probability of pseudo-conformity. In the baseline scenario, individuals were assumed to have complete behavioral flexibility, allowing them to adopt any exploration level between 0 and 1. The same analyses were repeated under scenarios in which individuals experienced constraints on their behavioral flexibility.

## Results

### Individuals are fully flexible

Across all 972 ecological scenarios examined, the proportion of groups that converged to the initial mean (ie exhibited pseudo-conformity) ranged from 0% to 86% (mean ± SEM: 0.282 ± 0.009). Moreover, whether through pseudo-conformity or adaptive phenotypic convergence, all group members converged after 80 learning steps towards the same trait value in almost all cases (mean ± SEM = 0.951 ± 0.005), meaning that individual differences in exploration were maintained in <5% of the ecological scenarios examined (Fig. 2a).

**Figure 2:**
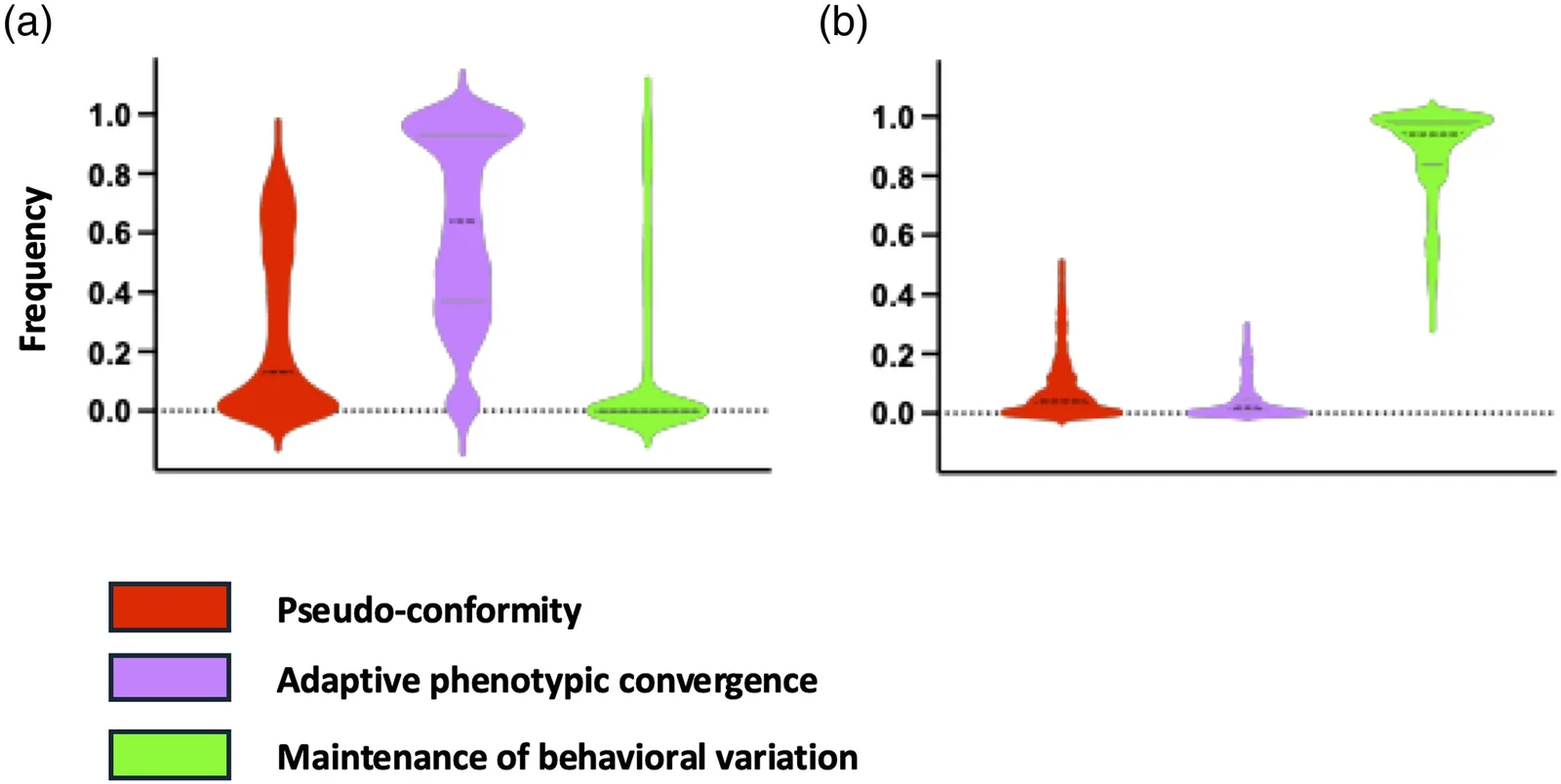
Violin plot showing the distribution of the mean proportion of groups that converged through pseudo-conformity (left “violon”), adaptive phenotypic convergence (middle “violon”) or maintained individual behavioral differences (right “violon”) across all 972 sets of parameter values. The width of each “violin” represents the density of data at a given value, the central line represents the median, and the outer lines the quartiles. Panel a illustrates the baseline scenario in which all group members are completely flexible (flex=1). Panel b illustrates the same ecological conditions but with constrained behavioral flexibility (flex=0.3).

As expected, ecological conditions strongly shaped the observed variation in the probability of pseudo-conformity. When both resource availability and the cost of exploration were low, groups typically converged toward an intermediate exploration level close to 0.5. Because this equilibrium was close to the expected initial mean exploration level, pseudo-conformity occurred frequently (Fig. 3). Increasing the cost of exploration shifted the adaptive equilibrium toward lower exploration levels, particularly in large groups, leading to a decline in pseudo-conformity and a corresponding increase in adaptive convergence toward a lower exploration optimum. By contrast, when resources were abundant, the observed pattern depended on group size (Figure S1): in small groups, the adaptive equilibrium corresponded to a high level of exploration regardless of the cost of exploration. Because this equilibrium was well above the groups’ initial mean exploration level, pseudo-conformity occurred only rarely. In contrast, in large groups, the equilibrium exploration level declined as the cost of exploration increased, reflecting the combined effects of stronger competition and higher exploration costs. Consequently, the probability of pseudo-conformity was highest at intermediate exploration costs, where the adaptive equilibrium most closely matched the groups’ initial mean exploration level.

**Figure 3:**
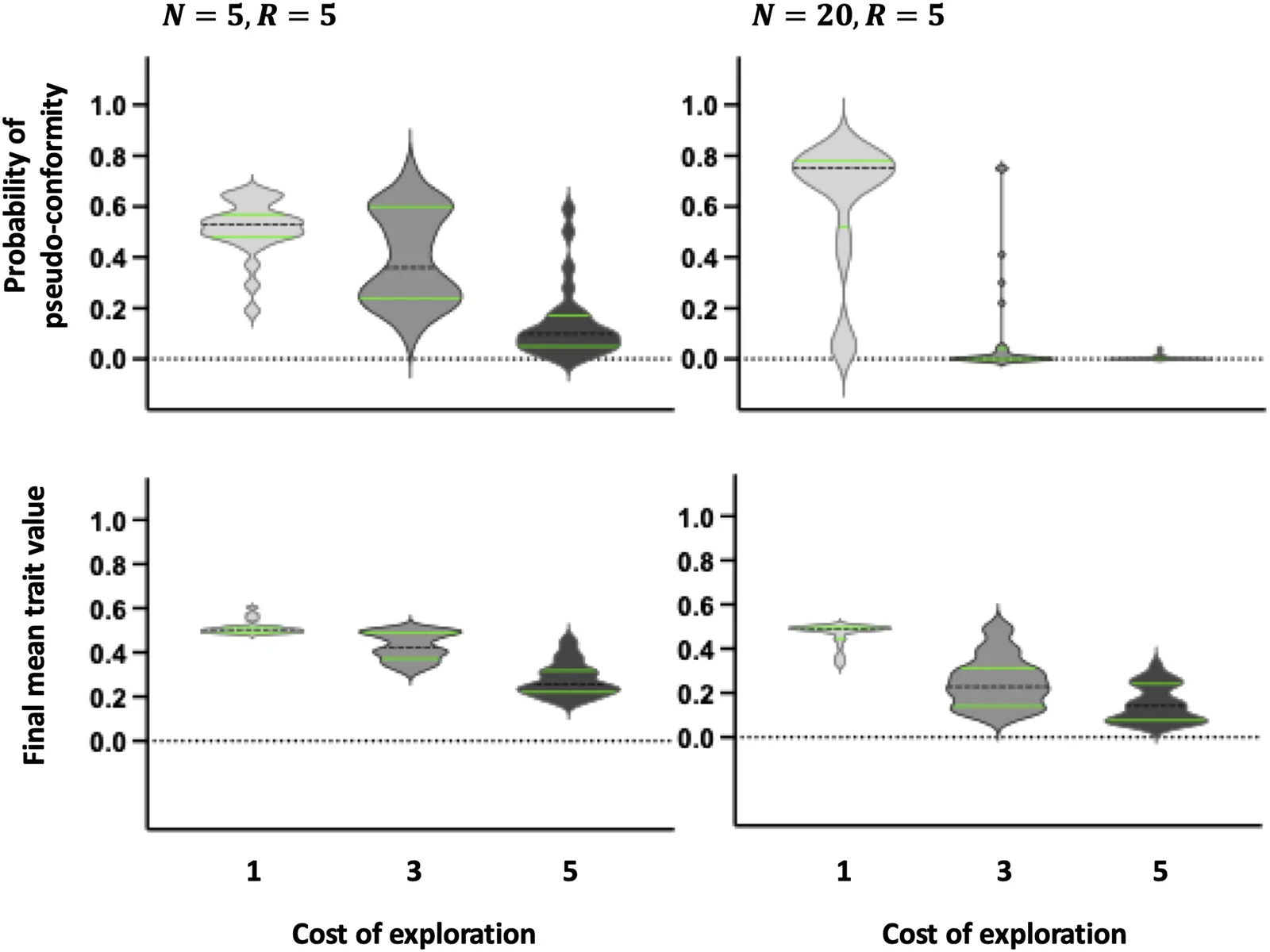
Violin plots showing the effects of the energetic cost of exploration on the proportion of groups exhibiting pseudo-conformity (top panels) and on the final mean exploration level after 80 learning steps of individual trial-and-error learning (bottom panels) across 2 group sizes, when resources are scarce and all individuals are fully flexible (flex=1). The width of each “violin” represents the density of data at a given value, the central dashed line represents the median, and the outer green lines the quartiles.

The probability of pseudo-conformity also depended on predation pressure (Fig. 4). Under low predation risk, pseudo-conformity remained rare regardless of the energetic cost of escaping a predator's attack. In contrast, under high predation risk, the likelihood of pseudo-conformity increased with the energetic cost of escaping an attack. Under these conditions, converging toward the group's initial mean behavior reduced the risk of being the target of a predator's attack, making behavioral alignment increasingly advantageous as the cost of escaping increased. Consequently, different groups converged toward their own initial mean exploration level rather than toward a common adaptive optimum, thereby preserving among-group variation in mean exploration following learning (Figure S2). The strength of the oddity effect yet had little influence on the probability of pseudo-conformity (Figure S3). Although a stronger oddity effect increased the selective advantage of matching the behavior of other group members, it did not determine whether groups converged toward their initial mean exploration level or toward a different adaptive equilibrium. Instead, under ecological conditions associated with a high risk of predation (ie, large groups and high predation pressure), a strong oddity effect substantially increased the proportion of groups exhibiting adaptive convergence toward a common phenotype (Figure S3).

**Figure 4:**
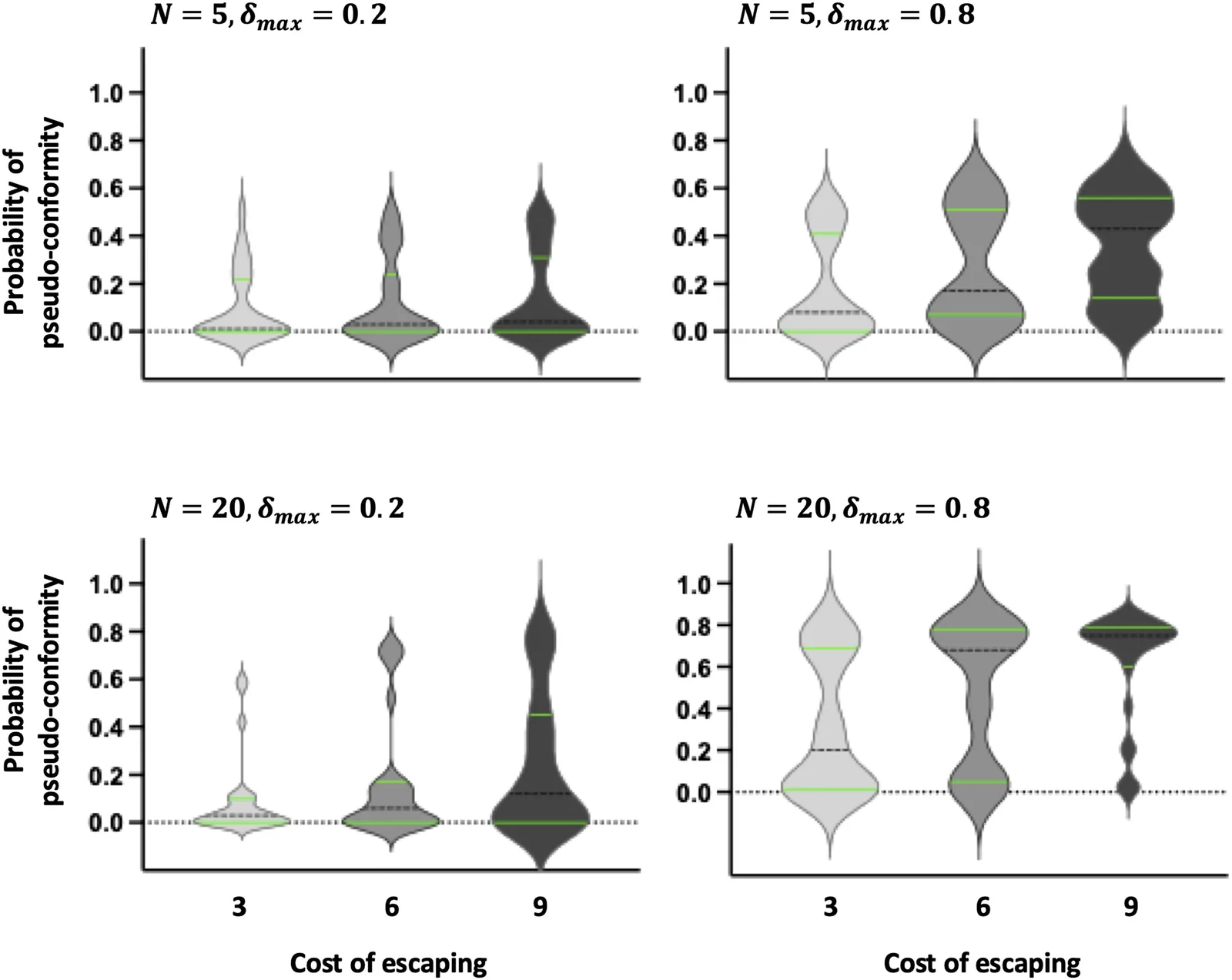
Violin plots showing the effect of the energetic cost of escaping an attack on the proportion of groups exhibiting pseudo-conformity across 4 ecological scenarios differing in group size and predation pressure, when all individuals are fully flexible (flex=1). The width of each “violin” represents the density of data at a given value, the central dashed line represents the median, and the outer green lines the quartiles.

In addition, for a given set of parameter values, the probability that the group converged toward the initial mean trait value could vary widely among replicates, hence suggesting that, beyond ecological factors, the social environment also shaped the likelihood of pseudo-conformity. Accordingly, under ecological conditions where pseudo-conformity occurred at intermediate frequencies (around 50%), its probability was generally positively associated with the initial mean trait value of group members (Table S1). Thus, individuals were more likely to converge toward the group mean exploration level expressed in isolation when groups were initially composed of highly exploratory individuals. This can be explained by the fact that if the group members exhibit low exploratory behavior, they contribute significantly to collective vigilance, thereby reducing the risk of a predator attack on the group. Under such conditions, individuals can thus afford to adopt a highly exploratory behavior compared with the rest of the group, to maximize the amount of energy acquired through resource exploitation without incurring a high risk of predation. Conversely, if the group members display high levels of exploratory behavior, and consequently contribute little to collective vigilance, the group becomes highly susceptible to predator attacks. Each group member then has no choice but to conform to the behavior of the group to reduce its likelihood of being targeted by the predator. This explanation is consistent with the fact that the influence of the group's initial mean exploration on the probability of pseudo-conformity varied across ecological conditions, mainly according to the predation risk and the energetic cost of escaping predator attacks (Fig. 5, Table S1). Specifically, under low predation risk, the initial exploration level of individuals tested in isolation had little effect on the probability of pseudo-conformity and, in some cases, pseudo-conformity was even more frequent in groups with lower initial exploration levels. By contrast, when predation risk was high, the probability of pseudo-conformity increased with the group's initial exploration level, indicating that highly exploratory groups were more likely to exhibit pseudo-conformity.

**Figure 5:**
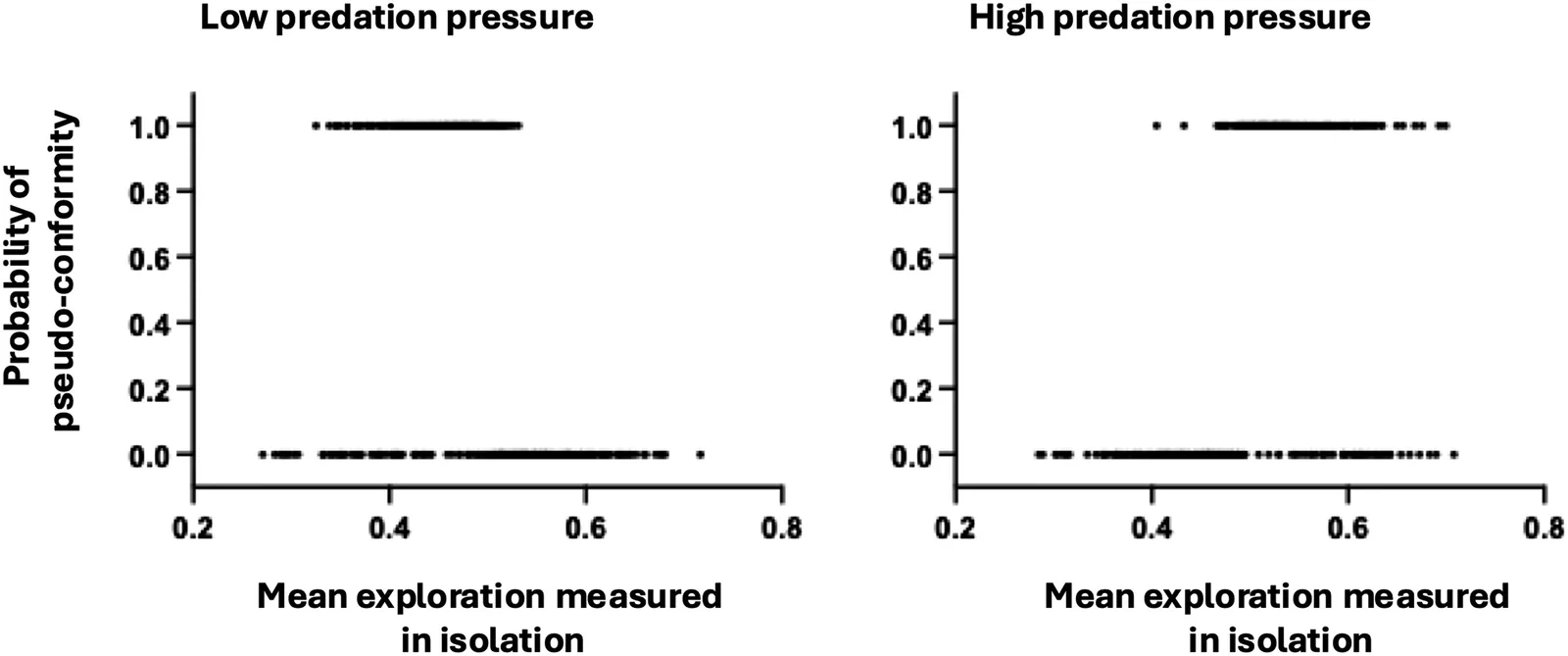
Logistic regression showing the relationship between the initial mean exploration level of group members in isolation and the probability of pseudo-conformity under low ($\partial_{\max}=0.2$) and high ($\partial_{\max}=0.8$) predation pressure, assuming unrestricted behavioral flexibility (flex=1).

### Individuals are limited in their flexibility

When individual flexibility was constrained, variation in exploratory behavior was maintained across most ecological scenarios (mean ± SEM: 0.844 ± 0.005, Fig. 2b). Despite this, the probability of pseudo-conformity still varied widely among ecological conditions and ranged from 0% to 47% (mean ± SEM: 0.102 ± 0.003), whereas adaptive convergence was extremely rare. Overall, larger groups were less likely than smaller groups to exhibit pseudo-conformity (Figures S4 and S5), because convergence toward the initial group mean required coordinated adjustments among a greater number of individuals. Yet, as group size increased, it became more likely that some individuals initially expressed exploration levels sufficiently different from the group mean that they could not reach it within their restricted range of behavioral adjustment. Consistent with this effect, the probability of pseudo-conformity was highest under low resource availability and in small groups, particularly when the energetic cost of exploration was low (Figure S4). In contrast, pseudo-conformity remained rare when resources were abundant or when groups were large despite limited resource availability, and its occurrence was largely unaffected by variation in the energetic cost of exploration under these conditions (Figure S4). As in the previous scenario with unrestricted behavioral flexibility, groups were also more likely to converge toward their initial mean exploration level when both predation risk and the energetic cost of escaping a predator attack were high (Figure S5).

Finally, under ecological conditions where pseudo-conformity occurred at an intermediate frequency (∼20% of groups), the initial mean exploration level was no longer a significant predictor of the probability of pseudo-conformity when considered alone. Instead, its effect became apparent only after accounting for the initial variance in exploration. This result is expected because, under limited behavioral flexibility, convergence toward the initial group mean depends not only on the mean exploration level of individuals in isolation but also on whether individuals are behaviorally similar enough to reach this value within their restricted range of behavioral adjustment. Moreover, the effect of the initial mean exploration level on the probability of pseudo-conformity varied across ecological conditions, primarily according to the energetic cost of escaping predator attacks (Figure S6, Table S2). Specifically, groups composed of highly exploratory individuals, which are more susceptible to predator detection, were more likely to converge toward a common phenotype through pseudo-conformity, particularly when escaping predator attacks was energetically costly.

## Discussion

The present study shows that conformist-like patterns can emerge solely from individual learning dynamics, without requiring individuals to assess the relative prevalence of behavioral variants or actively copy the behavior of their group mates. Instead, pseudo-conformity emerged as an indirect consequence of individuals adjusting their behavior through trial-and-error learning based on the tradeoff between competition and predation. This finding challenges the assumption that phenotypic convergence necessarily reflects social conformity mechanisms and highlights how similar population-level patterns can emerge from individual-level decision-making processes. In addition, even though numerous experimental studies have shown that animals adjust their behavior in social contexts to align with that of others, most have focused on comparisons between solitary individuals and individuals in the presence of conspecifics (typically a single conspecific). As a result, how ecological conditions and group composition jointly influence the emergence of conformity in larger groups remains poorly understood. The present study therefore provides a theoretical framework for identifying the ecological, social and individual conditions under which conformity-like patterns can emerge in the absence of conformist social learning.

Specifically, when behavioral flexibility was constrained, I found that behavioral variation was maintained across most ecological contexts because individuals were not always able to reach either the initial mean trait value or the ecological optimum. By contrast, when individuals were fully flexible, groups almost invariably converged toward a common phenotype, either because individuals converged toward their initial group mean (pseudo-conformity) or because ecological conditions favored convergence toward another adaptive phenotype. These findings suggest that cognitive or physiological constraints on behavioral flexibility may play a key role in maintaining behavioral diversity within populations (Wolf et al. 2008; Dubois et al. 2010). Furthermore, the emergence of pseudo-conformity was not a universal outcome of individual learning, but depended strongly on ecological, social and individual conditions. More generally, pseudo-conformity emerged whenever the ecological costs of deviating from the group's behavioral strategy outweighed the competitive benefits of expressing a different phenotype. Thus, when predation pressure was low, pseudo-conformity was generally rare, because individuals could increase their energetic gain by expressing higher exploration levels than their group mates, without incurring substantial costs associated with increased predator vulnerability. In contrast, under high predation risk, the probability of pseudo-conformity increased, particularly when escaping a predator attack imposed a large energetic cost. This prediction is supported by several empirical studies that have shown that behavioral variation among individuals tends to be greater in predator-free environments (Endler 1980; Theodorakis 1989; Nosil and Crespi 2006; Croft et al. 2009). Because larger groups tend to be more conspicuous to predators, the cost of deviation should also be greater in bigger groups, which should promote conformity (Webster and Ward 2011). Yet, empirical evidence for this relationship remains limited and inconsistent: while some studies have reported stronger conformity in larger groups under certain ecological conditions (Day et al. 2001), others have found the opposite trend (Hellström et al. 2011). In line with these inconsistencies, the present results show that increasing group size can either enhance or reduce the likelihood of pseudo-conformity, depending on ecological conditions. This occurs because larger groups face a higher risk of predator attack, which tends to favor pseudo-conformity, but also experience intensified competition, which promotes the expression of higher-than-average trait values. Therefore, the effect of group size on the probability of pseudo-conformity was modulated by both resource abundance and the energetic costs associated with elevated exploration levels. This finding underscores the importance of acknowledging that a given trait may exert contrasting effects on different components and demonstrates the need to simultaneously manipulate several factors to better understand how group size influences conformity. Moreover, the effect of group size, interestingly, depended strongly on behavioral flexibility. When individuals were fully flexible, large groups could more readily converge toward a shared phenotype because all individuals could adjust their behavior toward either the social mean or an adaptive optimum. In contrast, when flexibility was constrained, large groups were less likely to exhibit pseudo-conformity because convergence required coordinated adjustments among a greater number of individuals.

Beyond ecological conditions, pseudo-conformity was influenced by the composition of the group. Specifically, groups with initially high mean exploration were generally more likely to exhibit pseudo-conformity, probably because reduced collective vigilance increased predation risk and consequently the benefits of behavioral alignment. Conversely, when group members initially expressed low exploration and maintained high vigilance, the selective pressure for convergence weakened, allowing individuals to increase their own exploration and gain a competitive advantage. Importantly, however, this relationship depended on ecological conditions. The effect of the initial group phenotype became stronger as predation risk and the energetic costs of escaping attacks increased, indicating that the direction of behavioral adjustment emerges from the interaction between ecological constraints and social context rather than from either factor alone. Consistent with this prediction, previous empirical studies have reported that conformity-like adjustments may involve either less exploratory individuals increasing their exploration (King et al. 2015; Littlewood et al. 2021) or, conversely, highly exploratory individuals reducing exploration (Thomson et al. 2016; Fürtbauer and Fry 2018).

Taken together, these results suggest that behavioral convergence should not automatically be interpreted as evidence for a single evolutionary process. Instead, pseudo-conformity may arise through 2 fundamentally different pathways. First, convergence toward the group mean may simply emerge because individuals independently optimize their behavior under the same ecological constraints. In this case, pseudo-conformity is only observed when the initial group mean happens to coincide with the local adaptive optimum and all groups experiencing similar ecological conditions are expected to converge toward the same adaptive phenotype. Alternatively, pseudo-conformity may arise because the ecological costs associated with behavioral deviation make alignment with the group phenotype selectively advantageous. Under this scenario, groups with different initial compositions may each converge toward distinct behavioral norms despite experiencing similar ecological conditions. Consequently, these alternative pathways have markedly different evolutionary implications. Although both generate conformity-like patterns and reduce behavioral variation within groups, only the latter has the potential to maintain behavioral diversity at the population level by allowing different subgroups to stabilize around different behavioral phenotypes.

Distinguishing between these 2 pathways is therefore essential as the evolutionary consequences of behavioral convergence depend on the mechanisms generating it. Loss of behavioral diversity is generally assumed to reduce a population's capacity to respond to environmental changes (Wolf and Weissing 2012). However, convergence toward the group mean does not necessarily imply a loss of adaptive potential. When behavioral alignment itself is favored, populations structured into partially isolated social groups may retain substantial behavioral diversity because each subgroup converges toward its own locally stable behavioral equilibrium. By contrast, when convergence simply reflects optimization toward a common ecological optimum, behavioral variation is expected to erode both within and among groups. These contrasting predictions emphasize the importance of considering social structure (Aplin et al. 2013; Krause et al. 2015) when evaluating the ecological and evolutionary consequences of behavioral convergence. Finally, our results question the ecological relevance of behavioral traits measured in isolation, as individual behavior expressed alone may poorly predict behavior expressed in natural social environments (Brand et al. 2022). Future studies should therefore quantify behavioral variation under ecologically realistic social conditions, where interactions occur among naturally formed groups rather than isolated individuals.

## Supplementary Material

arag122_Supplementary_Data

## Data Availability

Analyses reported in this article can be reproduced using the data provided by Dubois (2026).
