# Supplementary material for: The illusion of conformity: when independent learning mimics social influence
Source: Behav Ecol. 2026 Oct 16;37(6):arag122. doi: 10.1093/beheco/arag122 (PMC13673270; doi:10.1093/beheco/arag122)
Supplement: Supplementary file 1 [file arag122_supplementary_data.zip › Supplementary material.docx]

**Supplementary material**

**The illusion of conformity: when independent learning mimics social influence**

**Figure S1**. Violin plots showing the effects of the energetic cost of exploration on the proportion of groups exhibiting pseudo-conformity (top panels) and on the final mean exploration level after 80 learning steps of individual trial-and-error learning (bottom panels) across two group sizes, when resources are abundant and all individuals are fully flexible ($flex=1$). The width of each ‘violin’ represents the density of data at a given value, the central dashed grey line represents the median, and the outer green lines the quartiles.

**Figure S2**. Violin plots showing the effect of the energetic cost of escaping an attack on the final mean exploration level after 80 learning steps of individual trial-and-error learning, across four ecological scenarios differing in group size and predation pressure, when all individuals are fully flexible ($flex=1$). The width of each ‘violin’ represents the density of data at a given value, the central grey dashed line represents the median, and the outer green lines the quartiles.

**Figure S3**. Violin plots showing how the strength of the oddity effect influences the proportion of groups that converged to a common exploration value through either pseudo-conformity (top panels) or adaptative convergence (bottom panels) under low ($\delta_{max}=0.2$) or high ($\delta_{max}=0.8$) predation pressure, in large groups where all individuals are fully flexible ($flex=1$). The width of each ‘violin’ represents the density of data at a given value, the central dashed grey line represents the median, and the outer green lines the quartiles.

**Figure S4.** Violin plots showing the effect of the cost of exploration on the proportion of groups exhibiting pseudo-conformity across four ecological scenarios differing in group size and resource availability, when all individuals are constrained in their flexibility ($flex=0.3$). The width of each ‘violin’ represents the density of data at a given value, the central grey dashed line represents the median, and the outer green lines the quartiles.

**Figure S5.** Violin plots showing the effect of the energetic cost of escaping an attack on the proportion of groups exhibiting pseudo-conformity across four ecological scenarios differing in group size and predation pressure, when all individuals are constrained in their flexibility ($flex=0.3$). The width of each ‘violin’ represents the density of data at a given value, the central grey dashed line represents the median, and the outer green lines the quartiles.

**Table S1**. Results of binary logistic regressions testing the effect of the mean exploration level of individuals in isolation on the probability of pseudo-conformity. Values, ranked from highest to lowest, are regression coefficients (b ± SE) based on 100 replicated datasets. Significant coefficients are shown in bold. The 30 ecological scenarios (parameter values are provided in the Table) were chosen because they yielded an intermediate frequency of pseudo-conformity, with 48–52% of groups converging to the initial mean exploration level, when individuals were fully flexible.

| $N$ | $R$ | $\delta_{max}$ | $c_{e}$ | $c_{m}$ | $odd$ | $b$ | SE |
| --- | --- | --- | --- | --- | --- | --- | --- |
| 20 | 35 | 0.5 | 9 | 1 | 0.6 | **18.97** | **4.57** |
| 20 | 20 | 0.5 | 6 | 1 | 0.1 | **17.15** | **4.41** |
| 15 | 35 | 0.8 | 9 | 1 | 1.1 | **14.06** | **3.63** |
| 10 | 20 | 0.2 | 3 | 3 | 1.1 | **13.69** | **3.37** |
| 10 | 20 | 0.5 | 3 | 3 | 0.1 | **12.34** | **3.02** |
| 15 | 20 | 0.8 | 6 | 1 | 0.1 | **10.01** | **3.26** |
| 5 | 20 | 0.2 | 9 | 5 | 1.1 | **8.26** | **2.08** |
| 10 | 35 | 0.8 | 3 | 5 | 1.1 | **7.78** | **2.6** |
| 5 | 20 | 0.5 | 6 | 5 | 0.1 | **6.67** | **1.99** |
| 10 | 20 | 0.2 | 9 | 3 | 0.1 | 6.15 | 2.27 |
| 10 | 35 | 0.5 | 6 | 5 | 0.6 | **6.05** | **3.07** |
| 5 | 5 | 0.8 | 3 | 1 | 0.1 | **5.50** | **1.87** |
| 10 | 20 | 0.8 | 3 | 3 | 0.1 | 4.92 | 2.59 |
| 5 | 5 | 0.2 | 6 | 1 | 1.1 | **3.71** | **1.63** |
| 10 | 20 | 0.2 | 9 | 3 | 0.6 | 3.36 | 2.36 |
| 5 | 20 | 0.5 | 9 | 5 | 0.6 | 2.09 | 1.92 |
| 5 | 5 | 0.8 | 3 | 1 | 1.1 | 1.69 | 1.55 |
| 10 | 5 | 0.5 | 9 | 1 | 0.6 | 0.93 | 2.41 |
| 10 | 5 | 0.8 | 9 | 1 | 1.1 | -0.08 | 2.53 |
| 5 | 5 | 0.8 | 9 | 1 | 0.1 | -0.10 | 1.44 |
| 10 | 5 | 0.2 | 6 | 1 | 0.1 | -2.31 | 2.39 |
| 5 | 20 | 0.8 | 9 | 5 | 0.6 | -2.94 | 1.92 |
| 5 | 5 | 0.8 | 3 | 3 | 0.6 | **-2.98** | **1.43** |
| 15 | 35 | 0.2 | 9 | 5 | 0.1 | -4.97 | 2.65 |
| 15 | 35 | 0.5 | 3 | 5 | 0.6 | **-8.35** | **3.10** |
| 15 | 35 | 0.5 | 3 | 5 | 0.6 | **-8.94** | **3.37** |
| 15 | 35 | 0.5 | 3 | 5 | 0.6 | **-9.60** | **3.04** |
| 15 | 5 | 0.2 | 6 | 1 | 0.1 | **-16.08** | **3.98** |
| 15 | 20 | 0.2 | 3 | 3 | 0.6 | **-21.27** | **4.79** |
| 20 | 5 | 0.2 | 6 | 1 | 1.1 | **-29.37** | **6.33** |

**Table S2**. Results of binary logistic regressions testing the effect of the mean exploration level of individuals in isolation on the probability of pseudo-conformity. Values, ranked from the highest to lowest, are regression coefficients (b ± SE) based on 100 replicated datasets. Significant coefficients are shown in bold. The 20 ecological scenarios (parameter values are provided in the Table) were chosen because they yielded an intermediate frequency of pseudo-conformity, with 10–40% of groups converging to the initial mean exploration level, when individuals were restricted in their behavioural flexibility.

| $N$ | $R$ | $\delta_{max}$ | $c_{e}$ | $c_{m}$ | $odd$ | $b$ | SE |
| --- | --- | --- | --- | --- | --- | --- | --- |
| 15 | 35 | 0.8 | 6 | 5 | 0.6 | **10.3** | **2.98** |
| 20 | 35 | 0.8 | 9 | 3 | 0.6 | 7.62 | 4.77 |
| 5 | 20 | 0.8 | 9 | 3 | 0.6 | **5.42** | **1.78** |
| 5 | 20 | 0.5 | 9 | 5 | 0.6 | **4.87** | **1.93** |
| 10 | 5 | 0.8 | 3 | 1 | 0.1 | 4.28 | 3.12 |
| 10 | 35 | 0.5 | 9 | 5 | 1.1 | 3.12 | 2.82 |
| 10 | 20 | 0.5 | 9 | 5 | 1.1 | 2.81 | 3.36 |
| 15 | 5 | 0.5 | 6 | 1 | 0.1 | 2.66 | 3.45 |
| 5 | 20 | 0.8 | 6 | 3 | 0.1 | 2.11 | 2.37 |
| 10 | 35 | 0.8 | 9 | 5 | 1.1 | 1.64 | 2.57 |
| 10 | 5 | 0.8 | 6 | 1 | 0.1 | 1.55 | 2.69 |
| 5 | 5 | 0.2 | 9 | 1 | 0.1 | 1.13 | 1.12 |
| 5 | 20 | 0.5 | 3 | 5 | 0.1 | -0.23 | 1.87 |
| 5 | 5 | 0.5 | 6 | 3 | 0.1 | -1.32 | 1.57 |
| 10 | 35 | 0.5 | 9 | 5 | 0.6 | -1.88 | 2.58 |
| 15 | 5 | 0.8 | 6 | 3 | 0.6 | -2.92 | 3.53 |
| 20 | 20 | 0.2 | 9 | 1 | 0.6 | -5.03 | 5.04 |
| 15 | 20 | 0.2 | 9 | 3 | 1.1 | -6.99 | 3.72 |
| 5 | 5 | 0.5 | 3 | 3 | 0.1 | **-9.23** | **2.47** |
| 15 | 20 | 0.8 | 9 | 1 | 1.1 | **-11.23** | **5.48** |
